# Isolation of Nb_2_Se_9_ Molecular Chain from Bulk One-Dimensional Crystal by Liquid Exfoliation

**DOI:** 10.3390/nano8100794

**Published:** 2018-10-06

**Authors:** Sudong Chae, Akhtar J. Siddiqa, Seungbae Oh, Bum Jun Kim, Kyung Hwan Choi, Woo-Sung Jang, Young-Min Kim, Hak Ki Yu, Jae-Young Choi

**Affiliations:** 1 School of Advanced Materials Science & Engineering, Sungkyunkwan University, Suwon 16419, Korea; csd5432@gmail.com (S.C.); akhtariitkgp@gmail.com (A.J.S); nysbo0219@gmail.com (S.O.); 2 SKKU Advanced Institute of Nanotechnology (SAINT), Sungkyunkwan University, Suwon 16419, Korea; kbj454@gmail.com (B.J.K.); chhcc12@gmail.com (K.H.C.); 3 Department of Energy Science, Sungkyunkwan University, Suwon 16419, Korea; aiw23@naver.com (W.-S.J.); youngmk@skku.edu (Y.-M.K.); 4 Center for Integrated Nanostructure Physics, Institute for Basic Science (IBS), Suwon 16419, Korea; 5 Department of Materials Science and Engineering & Dept. of Energy Systems Research, Ajou University, Suwon 16499, Korea

**Keywords:** 1D materials, Nb_2_Se_9_, liquid exfoliation, solvent dispersion

## Abstract

The optimum solvent for Nb_2_Se_9_ dispersion, which is a new type of one dimensional (1D) material, is investigated. Among several solvents (16 solvents in total), strong dispersion was observed in benzyl alcohol, isopropyl alcohol, isobutyl alcohol, and diacetone alcohol, which have medium dielectric constants in the range of 10 to 30 and surface tension in the range of 25 to 35 mJ m^−2^. 1D Nb_2_Se_9_ chains, whose size is less than 10 nm, are well dispersed and it is possible to disperse mono-chains of 1 nm or less in a specific dispersion region. The 1D unit chain with dangling bond free surface and high volume to area ratio is expected to be used in applications that utilize the surface of the material. Such dispersion is an important first step towards various potential applications and is an indispensable scientific goal for the practical applications of Nb_2_Se_9_.

## 1. Introduction

Among the great variety of nanomaterials available, one-dimensional (1D) materials, including nanowires and carbon nanotubes (CNTs), have been extensively studied due to their remarkable physical and chemical properties such as high carrier mobility [1,2,3], high chemical stability [4], high mechanical strength [4,5], and large surface area [5]. These unique properties allow 1D materials to be applied as building blocks for numerous applications, such as field-effect transistors (FETs), sensors, and nanocomposites.

Other types of 1D materials, such as LiMo_3_Se_3_ [6,7,8,9,10], Mo_6_S_3_I_6_ [11,12,13,14], and Mo_6_S_4.5_I_4.5_ [15,16] have been investigated by several researchers. These materials were obtained by exfoliating 1D bulk crystals into nanowires or molecular chains, because there exists weak van der Waals (vdW) interactions or ionic bonds between the unit chains in 1D bulk crystals, similar to those observed in 2D materials such as graphene and 2D transition metal dichalcogenides (TMDCs). Thus, these 1D bulk crystals provide a way to easily obtain nanowires or inorganic molecular chains less than 1 nm in diameter. When isolated from bulk crystals, molecular chains have unique surface characteristics. LiMo_3_Se_3_ has a negative charge on the chain surface due to ionic interactions between Li^+^ ions and Mo_3_Se_3_^−^chains [17] Mo_6_S_3_I_6_ and Mo_6_S_4.5_I_4.5_, on the other hand, undergo vdW interactions and thus dangling bonds exist on their chains, similar to the case in graphene and transition metal dichalcogenides(TMDC). Due to these structural features [11], they have unique physical and chemical properties, which leads to many useful applications such as transistor [12,18,19,20], sensor [21], composite [13] and solar cell [22,23]. In addition, new 1D bulk materials, Sb_2_S_3_ [24] and Sb_2_Se_3_ [25,26], have been reported to have excellent optoelectronic properties due to the absence of dangling bonds on their chain surfaces.

Recently, we demonstrated the preparation of new 1D bulk crystals of Nb_2_Se_9._ The crystals were synthesized by a solid-state reaction and could be reproduced in large quantities; furthermore, they were stable in air (these properties are essential characteristics for the subsequent processes). Therefore, it is important to obtain nanowires or molecular chains from bulk 1D crystals. For example, CNTs, which are initially a bundle of unit tubes put together by vdW attraction forces, were exfoliated to yield individual tubes which can be used in many applications [27,28,29]. Therefore, exfoliation is an important method for fabricating 1D structures and might potentially be applied on Nb_2_Se_9_. Although there are a few reports of the synthesis of Nb_2_Se_9_ bulk materials reported 30 to 40 years ago [30,31,32], few studies have been done to obtain the unit chain of Nb_2_Se_9_ through liquid exfoliation. In order to obtain the unit chain of Nb_2_Se_9_ in the liquid phase, it is possible to apply the previous approaches used for the exfoliation or dispersion of nanomaterials. Typically, approaches to design solvent combinations [33,34] or dispersants [35,36] have been utilized and information on the surface properties of materials such as surface tension, dielectric constant, solubility parameter can be useful for these strategies. Herein, we exfoliated Nb_2_Se_9_ bulk crystals in various solvents because this method is simple and can result in large amounts of the samples, and provide the basic information of the material’s surface for the further exfoliation strategy. In this study, we tried to find an optimal solvent for the exfoliation process and also verify whether single molecular chains can be obtained from the said solvent. 

## 2. Materials and Methods 

### 2.1. Synthesis

Nb_2_Se_9_ was produced from elemental powders of niobium (325 mesh, 99.5%, Sigma-Aldrich, St. Louis, MO, USA) and selenium (99+%, Alfa Aesar, Haverhill, MA, USA) using a flux method. 2.15 mmol of Nb powder (0.2 g) and 430 mmol of Se powder (34 g) were thoroughly mixed and sealed in a quartz tube designed with a compartment and neck in which unreacted Se flux collects after reaction. The evacuated quartz tube was placed in a box furnace and heated to 800 °C for 72 h (at 5.5 °C h^−1^) and then cooled (at 10 °C h^−1^). After cooling, the quartz tube was turned upside down and heated again to 250 °C for 12 h in order to drop the unreacted flux onto the other side of the tube. Finally, residual Se was sublimed in a low-pressure tube furnace at 250 °C for 24 h under Ar flow (100 sccm). The resulting material was gray needle-shaped crystals.

### 2.2. Dispersion

Nb_2_Se_9_ crystals were dispersed in different solvents by ultrasonication. Firstly, vials were filled with 10 mg of Nb_2_Se_9_ and 10 mL of the chosen solvent and sonicated for 5 min in a probe sonicator (VC 505, Sonics & Materials, Inc., Newtown, CT, USA) in order to break down any large crystals. After the pre-sonication process, bath sonication (B2005S-68K, 68 kHz, 200 W, KODO Technical, Hwaseong, South Korea) was conducted for 3 h. Later, the solutions were centrifuged at 6000 rpm for 10 min to remove insufficiently exfoliated chains. Five milliliters of the supernatant solution was used for further analysis. 

### 2.3. Characterization

X-ray diffraction (XRD) patterns of Nb_2_Se_9_ crystals were obtained by powder XRD (Mac Science, M18XHF22, Tokyo, Japan) with Cu-Kα radiation (λ = 0.154 nm). Field-emission scanning electron microscopy (FE-SEM, Hitachi, S-4300SE, Chiyoda, Tokyo, Japan) was performed to examine the morphological characteristics of the Nb_2_Se_9_ crystals. An aberration-corrected scanning transmission electron microscope (STEM, JEM ARM 200F, JEOL, Tokyo, Japan) operating at an acceleration voltage of 80 kV was used for further morphological analysis. For sample preparation, drop casting was carried out on a graphene-coated Quantifoil TEM grid. The concentration of the dispersion solution was confirmed by Inductively coupled plasma mass spectrometry (ICP-MS, Agilent 7500, Agilent Technologies Inc., Santa Clara, CA, USA). To evaluate the morphology of the exfoliated Nb_2_Se_9_, Atomic force Microscopy (AFM, Park systems, NX10, Suwon, South Korea) was performed on Nb_2_Se_9_ spin-coated SiO_2_/Si wafers in the non-contact mode.

## 3. Result and Discussion

**Figure 1:**
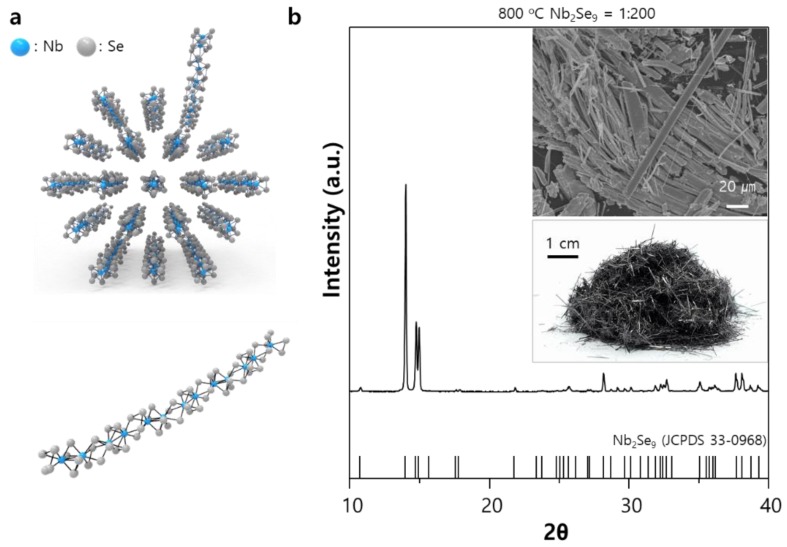
(**a**) Schematic illustration of the crystal structure and exfoliation process of Nb_2_Se_9_; (**b**) X-ray diffraction (XRD) pattern of Nb_2_Se_9_ crystals (inset shows the scanning electron microscopy (SEM) of Nb_2_Se_9_ crystals (left) and STEM image of the exfoliated Nb_2_Se_9_ (right)).

The structure of a Nb_2_Se_9_ chain is described as a 1D molecular chain of niobium (Nb) atoms linearly connected with each other and selenium (Se) atoms decorated on the outside of niobium atoms (belonging to point group $\bar{1}$, space group P$\bar{1}$. Nb is located in the octahedral site constructed by Se atoms. See top of Figure 1a). During the dispersion process, single molecular chains can be exfoliated from the bulk crystal due to weak vdW interactions between the chains (bottom of Figure 1a). Single crystalline Nb_2_Se_9_ was synthesized by chemical reactions between Nb and Se in the quartz ample. To prevent the formation of another niobium selenide compounds such as NbSe_3_ [37] and NbSe_2_ [38], we used high niobium to selenium ratio of 1:200. Only the Nb_2_Se_9_ and Se phase can be formed in the composition, and the selenium phase can be selectively removed through heat treatment [39]. When the Nb-Se mixture at 700–800 °C was cooled down to room temperature, dark gray needle-shaped crystals were formed (see the experimental section for details) and X-ray diffraction (XRD) analysis confirmed that the material contained a highly crystalline Nb_2_Se_9_ phase (JCPDS 33-0968) (Figure 1b). The inset of Figure 1b shows digital and scanning electron microscope (SEM) images of the Nb_2_Se_9_ crystals prepared in this study. It is observed that large needle-shaped Nb_2_Se_9_ crystals (length in the range of a few centimeters) were successfully grown. Additionally, some Nb_2_Se_9_ crystals are naturally exfoliated in the form of chains; consequently, the material synthesized in this study can be dispersed as 1D units if we can find a suitable solvent. 

**Figure 2:**
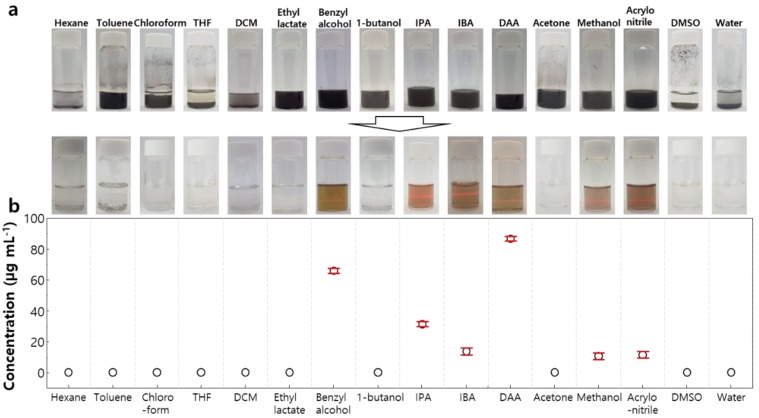
(**a**) Photographs of dispersion solutions after ultrasonication (top) and separated supernatant after centrifugation exhibiting a Tyndall effect (bottom); (**b**) Concentration of the Nb_2_Se_9_ dispersion solution depending on the solvent. Error bars represent standard deviations obtained from three measurements of the same sample.

The liquid exfoliation method is known to be insensitive to air and can potentially be scaled up to yield large quantities of the exfoliated material [40]. Since the organic solvents used in the solution process mainly have toxicity problems and sustainability issues, it is necessary to increase the process efficiency to minimize the use of solvents and to select less hazardous solvents [41,42]. In order to find the best exfoliation solvent for Nb_2_Se_9_ crystals, 16 common solvents with different dielectric constants and surface energies were studied (Table 1). In order to find the optimum solvents for exfoliation of Nb_2_Se_9_, various solvents with a broad dielectric constant of approximately 1 to 80 and a surface tension (mJ m^−2^) of 18 to 73, which are mainly used for the dispersion of nanomaterials [40,43,44]. Nb_2_Se_9_ particles were dispersed in each solvent by sonication and centrifuged to obtain a well dispersed supernatant fraction after removing large and un-exfoliated particles (see the experimental part for details). Digital photos of the dispersed solutions before and after centrifugation are shown in Figure 2a. A strong Tyndall effect (laser scattering due to nano-scale dispersion) was observed in dispersions in benzyl alcohol, isopropyl alcohol (IPA), isobutyl alcohol (IBA), and diacetone alcohol (DAA). These solvents are the sustainable green solvents that have no serious environmental, health, or safety hazards in the industry [45]. The concentration of Nb_2_Se_9_ was measured by inductively coupled plasma mass spectrometry (ICP-MS) and the concentration of Nb_2_Se_9_ was found to be high in the solvent exhibiting a strong Tyndall effect (Figure 2b). The top three solvents have a reproducible result with a standard deviation of less than 5%.

**Figure 3:**
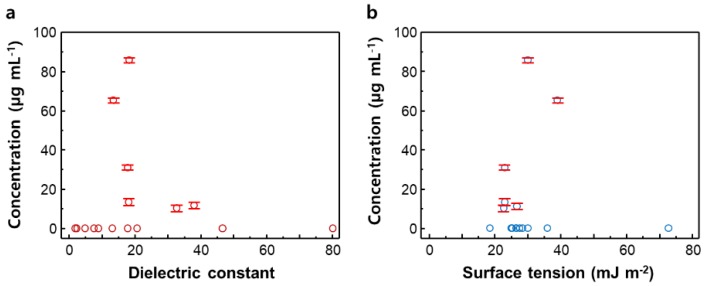
Concentration of the Nb_2_Se_9_ dispersion solution versus (**a**) dielectric constant and (**b**) surface tension Error bars represent standard deviations obtained from three measurements of the same sample.

In order to understand the dispersion characteristics of Nb_2_Se_9_ with respect to the solvent, the concentrations of the dispersions according to solvent characteristics (dielectric constant and surface energy) are plotted, as shown in in Figure 3. It can be seen that the dispersion concentration is high in solvents with a medium dielectric constant of 10 to 30 and surface tension in the range of 25 to 35 mJ m^−2^, which is similar to the dispersion behavior of 2D TMDCs. Dispersions studies of TMDCs in different solvents have shown that most TMDCs including MoS_2_, MoSe_2_, MoTe_2_, and NbSe_2_ are effectively dispersed in medium polar solvents with surface energy in the range of 30 to 40 mJ m^−2^ [30]. 1D Nb_2_Se_9_ is structurally similar to 2D TMDCs in that the chalcogenide atoms enclose a transition metal core and the outermost surface is composed of chalcogenide atoms. In detail, Nb_2_Se_9_ chains exhibit a structure in which the core Nb atom is surrounded by Se atoms, while the TMDC layer is a structure in which an inner transition layer is sandwiched between two chalcogenide atom sheets. Additionally, Nb_2_Se_9_ chains and TMDC sheets exhibit similar weak vdW interactions between the chain and layer. Thus, Nb_2_Se_9_ shows a dispersion tendency similar to that of TMDCs as both materials have similar surface structure and interaction forces between the unit chains and sheets. 

**Figure 4:**
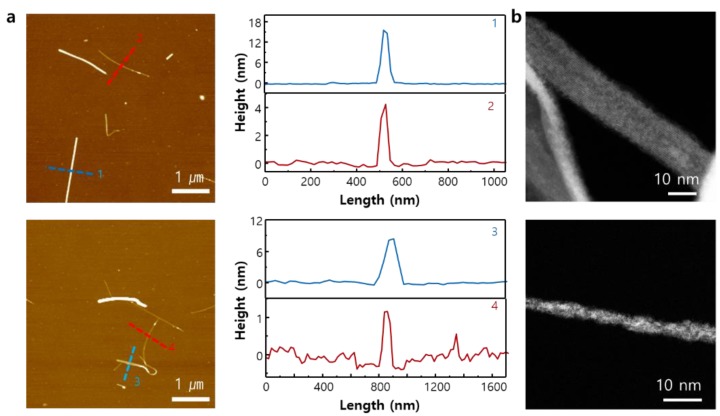
(**a**) Atomic force microscopy (AFM) image of the exfoliated Nb_2_Se_9_ nanowires on Si/SiO_2_ wafers; the height profiles are shown along each dashed line; (**b**) Annular dark-field (ADF)-STEM image of the exfoliated Nb_2_Se_9_ nanowires.

Dispersed Nb_2_Se_9_ particles in DAA were spin-coated on SiO_2_/Si substrates and the size of the nano-chains was analyzed by atomic force microscopy (AFM) and transmission electron microscopy (TEM) (see Figure 4a,b). The 1D chains of Nb_2_Se_9_ with sizes less than 10 nm are well dispersed (possible to disperse mono-chains to 1 nm or less in a specific dispersion region: line profile 4). Additionally, the aspect ratio (length/diameter) of Nb_2_Se_9_ chains is important in device fabrication and composite formation. The aspect ratio of Nb_2_Se_9_ chains dispersed in DAA is found to be about 542. It is expected that greater dispersion of 1D Nb_2_Se_9_ chains into atomic units will be possible after optimization of the dispersion solvent and the dispersion process. 

## 4. Conclusions

In summary, a novel 1D inorganic molecular chain material (Nb_2_Se_9_) was synthesized through chemical reaction between Nb and Se; 1D nano-sized (≤10 nm) Nb_2_Se_9_ molecular chains were successfully obtained by dispersion. Of the various solvents tested (total 16 solvents), strong dispersions were obtained with green solvents such as benzyl alcohol, isopropyl alcohol, isobutyl alcohol, and diacetone alcohol, whose dielectric constant was in the range of 10 to 30 and surface tension was in the range of 25 to 35 mJ m^−1^. The best results were obtained in a diacetone alcohol with a concentration of 85.83 µg mL^−1^. It can be enhanced through the additional design of solvent combination and dispersant. These results can provide the essential information of the material’s surface for design of the further exfoliation strategy, and can be recognized as an important step towards various potential applications of 1D Nb_2_Se_9_, such as transistors, sensors, composites, and solar cells.

## Figures and Tables

**Table 1:** Molecular structure, surface tension, and dielectric constant of the 16 selected solvents.

Solvent	Molecular Structure	Surface Tension (mJ m^−2^)	Dielectric Constant
Hexane	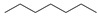	18.43	1.89
Toluene	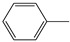	28.43	2.38
Chloroform	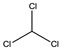	27.5	4.81
Tetrahydrofuran (THF)	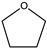	26.4	7.58
Dichloromethane (DCM)	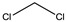	26.5	8.93
Ethyl lactate	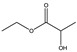	30	13.1
Benzyl alcohol	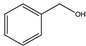	39	13.5
1-butanol	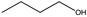	25	17.8
Isopropyl alcohol (IPA)	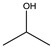	23	17.9
Isobutyl alcohol (IBA)	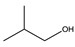	22.98	18.1
Diacetone alcohol (DAA)	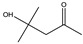	30	18.2
Acetone	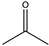	25.2	20.7
Methanol	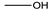	22.7	32.7
Acrylonitrile	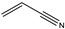	26.7	38
Dimethyl sulfoxide (DMSO)	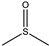	36	46.7
Water	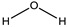	72.8	80.1
